# Prototheca-ID: a web-based application for molecular identification of Prototheca species

**DOI:** 10.1093/database/baab073

**Published:** 2021-11-13

**Authors:** Mikołaj Dziurzyński, Przemyslaw Decewicz, Mateusz Iskra, Zofia Bakuła, Tomasz Jagielski

**Affiliations:** Department of Environmental Microbiology and Biotechnology, Institute of Microbiology, Faculty of Biology, University of Warsaw, I. Miecznikowa 1, Warsaw 02-096, Poland; Department of Environmental Microbiology and Biotechnology, Institute of Microbiology, Faculty of Biology, University of Warsaw, I. Miecznikowa 1, Warsaw 02-096, Poland; Department of Medical Microbiology, Institute of Microbiology, Faculty of Biology, University of Warsaw, I. Miecznikowa 1, Warsaw 02-096, Poland; Department of Medical Microbiology, Institute of Microbiology, Faculty of Biology, University of Warsaw, I. Miecznikowa 1, Warsaw 02-096, Poland; Department of Medical Microbiology, Institute of Microbiology, Faculty of Biology, University of Warsaw, I. Miecznikowa 1, Warsaw 02-096, Poland

## Abstract

The genus *Prototheca* houses unicellular, achlorophyllous, yeast-like algae, widely distributed in the environment. Protothecae are the only known plants that have repeatedly been reported to infect vertebrates, including humans. Although rare, protothecosis can be clinically demanding, with an unpredictable and treatment-resistant behavior. Accurate identification of *Prototheca* species relies upon DNA sequence-based typing of the mitochondrially encoded *CYTB* gene. However, no bioinformatic tool for the processing and analyzing of protothecal sequence data exists. Moreover, currently available sequence databases suffer from a limited number of records and lack of or flawed sequence annotations, making *Prototheca* identification challenging and often inconclusive. This report introduces the Prototheca-ID, a user-friendly, web-based application providing fast and reliable speciation of *Prototheca* isolates. In addition, the application offers the users the possibility of depositing their sequences and associated metadata in a fully open Prototheca-ID database, developed to enhance research integrity and quality in the field of Protothecae and protothecosis.

**Database URL**: The Prototheca-ID application is available at https://prototheca-id.org

**Key Points**

- Prototheca-ID is a first, open-source, online toolbox for the molecular identification of *Prototheca* species.
- Prototheca-ID collects sequences of *Prototheca* cytochrome b (*CYTB*) and D1/D2 region of the large subunit of the rRNA genes—key markers for *Prototheca* spp. identification.
- Prototheca-ID contains 172 validated, high-quality sequences of 87 *Prototheca* strains, representing all currently known species, including their type strains.
- Prototheca-ID users can analyze their sequences through the ‘Analyze’ subpage, which provides the identification match, based on the highest alignment score, and generates a dendrogram to illustrate the phylogenetic relationships of the subject to members of the database.

## Introduction

*Prototheca* are unicellular, colorless, nonphotosynthetic microalgae, ubiquitously distributed in nature. Although they normally live a saprophytic lifestyle, occurring most abundantly in humid and organic-rich environments, they may, under certain conditions, act as opportunistic pathogens, causing a variety of pathologies in both animals and humans, collectively referred to as protothecosis (1, 2). *Prototheca* are the only known plants that have repeatedly been reported to infect vertebrates, including men. In animals, the disease most commonly affects dairy cattle, resulting in chronic, drug-resistant mastitis. Bovine mammary protothecosis has recently become an emerging health and economic problem to the veterinary sector worldwide (3–6). Next, to streptococci and staphylococci, *Prototheca* algae are the key mastitis pathogens. The prevalence of the protothecal disease ranges from 5% to nearly 17% among dairy herds across the world (3). Human protothecosis is a rare condition, with a total of 211 cases reported globally by 2017 (7). Notwithstanding, the incidence of the disease has been on the rise over the past two decades due to the growing population of elderly and otherwise immunocompromised individuals but also due to improved clinical awareness and recent technological advancements in the diagnostic approaches (7, 8). According to a new report updating the global caseload of human protothecosis, there have been a total of 335 cases by 2020 (Jagielski T. *et al.*, data unpublished).

The *Prototheca* taxonomy has long been controversial and subject to several revisions. The advent of molecular markers has greatly facilitated elucidating the phylogenetic relationships of the *Prototheca* algae, leading to considerable improvements in their identification.

Until recently, the only target for the molecular taxonomic approaches has been the ribosomal RNA gene cluster. Numerous studies have exploited sequence polymorphisms within the small- and large-subunit (SSU, LSU) rRNA genes and the internal transcribed spacer (ITS) region for investigating phylogenetic relatedness among *Prototheca* species and allied taxa and for developing new typing schemes to achieve fast and accurate identification of the algae (9).

However, the rDNA markers do not provide sufficient discriminatory power to effectively separate all *Prototheca* species currently recognized. What further impedes the use of rDNA markers is a high level of intraspecific and intrastrain sequence variation (9–11).

Recently, we have proposed the mitochondrially encoded *CYTB* gene as a new and powerful marker for diagnostics and phylogenetic studies of the *Prototheca* algae (9). The *CYTB* gene was shown superior to rDNA markers in terms of discriminatory capacity and technical feasibility (i.e. PCR amplification, sequencing and sequence analysis). Based on the *CYTB* gene marker, a new taxonomic classification system of the *Prototheca* algae has been established (12). Furthermore, a PCR-RFLP (polymerase chain reaction-restriction fragments length polymorphism) assay targeting sequence polymorphisms within the *CYTB* gene has been developed as a rapid and reliable means of *Prototheca* identification at the species level (9, 12). Given, however, that some *Protothecaciferrii* strains may produce PCR-RFLP patterns characteristic for *P. bovis*, to distinguish between the two species, direct sequencing of the *CYTB* gene is required (12). Consequently, sequencing of the *CYTB* gene provides the highest accuracy of *Prototheca* species delimitation. In fact, it is the only approach, currently known, allowing for unambiguous identification of all 15 *Prototheca* species described so far.

In a clinical setting, the use of *CYTB* gene-based typing, and *CYTB* sequencing in particular, is advocated as a new standard in diagnostics of protothecal infections in both human and animal hosts.

The success of any sequence-based taxonomic profiling depends critically on high-quality, well-annotated reference sequence databases. However, the contamination of publicly available sequence repositories with incorrectly annotated or otherwise poorly described sequences is quite common, leading to either misidentification or no identification result at all.

Currently, the largest collection of *Prototheca*-derived DNA sequences provides the GenBank database. As of 1 May 2021, GenBank contained 8948 *Prototheca*-related sequences with seven assemblies for only five *Prototheca* species. One thousand five hundred thirty-nine (17%) out of 8948 sequences were for the rDNA loci, including the ITS region. Only 98 (1%) sequences represented the *CYTB* gene, the bulk of which (87) was submitted by the authors of this communication. Being not actively curated by taxonomic specialists, GenBank often contains erroneous, outdated or misleading data, precluding the correct species identification. Moreover, GenBank suffers from the lack of rigorous data collection and structuring, potentially useful for comparative, analytical and interpretative purposes. These pitfalls have been recognized for many GenBank deposited *Prototheca* sequences. Given the growing prevalence of Protothecae, and the ongoing discovery of new species, databases such as GenBank are expected to be inflated by incorrectly or insufficiently annotated entries.

There is thus a need for an integrative and expert-curated database rendering a robust and reliable identification of *Prototheca* species.

To address this need, the Prototheca-ID project has been launched, introducing the Prototheca-ID web application, a freely accessible, easy-to-use toolbox designed for sequence-based, species-level identification of *Prototheca* isolates.

## Prototheca-ID application

The Prototheca-ID web-based application (https://prototheca-id.org) has a two-component construction. It comprises of a manually curated database of *Prototheca*-derived *CYTB* and LSU marker sequences and a species-level taxonomy analysis tool. The database contains a set of regularly updated and manually curated sequences of the two *Prototheca* markers, allowing for a fast and reliable identification of *Prototheca* isolates. Every sequence in the database is provided with metadata specifying the origin of sequence or the strain details, including its source of isolation, year of deposition, names of depositor(s) and appropriate reference if available. The users can not only search through the database or download its content, upon request, but are welcome to deposit their sequence(s), along with selected information on the strains so that the database can expand easily, increasing the accuracy of sequence matching and thus successful identification. A *Prototheca* sequence of *CYTB* or LSU coding regions will be considered for Prototheca-ID database if: (i) amplified using a high-fidelity polymerase and primers previously reported by Jagielski *et al.* (9), (ii) deposited in the Genbank database, (iii) the following sequence metadata are available: species name, strain number and Genbank accession number. The optional, yet recommended, metadata include: source type, country and year of isolation. This also enables investigators a more powerful exploration and analysis of the datasets.

**Figure 1.**
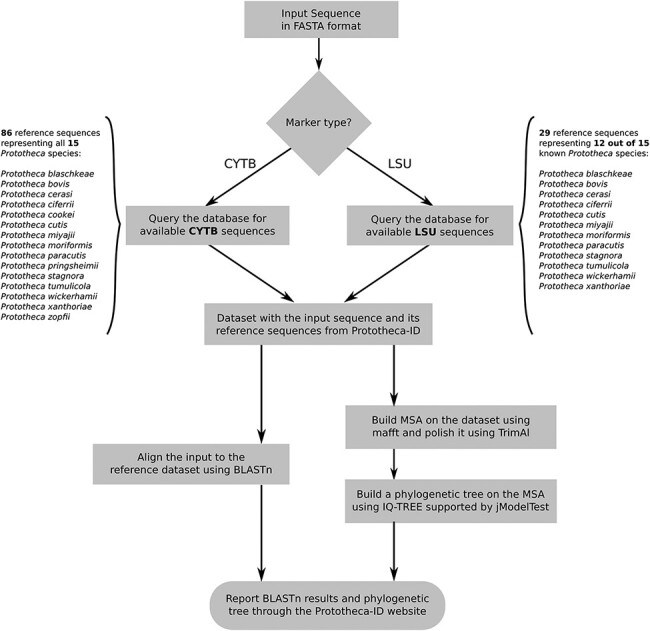
Flowchart depicting all steps of the Prototheca-ID sequence analysis pipeline.

The second module of the Prototheca-ID application consists of a sequence analysis and classification tool (Figure 1). A user can perform the identification of any of his *CYTB* or LSU nucleotide sequences in a fast and simple manner. The identification process is based on nucleotide sequence search with BLASTn against a selected group of reference genes, whereas the construction of the phylogenetic trees is based on the multiple sequence alignment (13). The initial search results are limited only to hits with at least 50% sequence identity and 50% query coverage. These results are subsequently used to perform multiple sequence alignment with mafft in auto mode (14, 15). The alignment is then curated with TrimAl by applying *-gapthreshold 0.3* and *-simthreshold 0.001* flags and passed to IQ-TREE (16, 17). The maximum likelihood phylogenetic tree is constructed based on the model selected with jModelTest (18). Branch support is calculated based on 1000 replicates of both ultrafast bootstrap and SH-like approximate likelihood ratio. At the end, the resulting phylogenetic tree is presented to the user through the phylotree.js library (https://github.com/veg/phylotree.js). The user can freely download the tree in the Newick format and perform further adjustments.

MSA—multiple sequence alignment.

## Conclusions

This paper reports on the development and implementation of a user-friendly, web-based analytical tool for fast and reliable identification of *Prototheca* species.

The core idea of the project was not only to deliver a simple and robust platform for *Prototheca* identification but also to establish a comprehensive, dedicated and fully open database of sequences of *Prototheca* isolates and isolate-related information, well-annotated, carefully verified and rigorously structured in a standardized and easily searchable manner.

Prototheca-ID was conceived as a regularly updated and continuously expanding database to include other taxonomic markers or markers associated with clinically and/or epidemiologically relevant phenotypes, such as drug resistance, virulence and transmissibility. The Prototheca-ID aims at integrating sequence data with provenance and phenotypic information on Protothecae, allowing scientists to study the biology of these organisms in the context of their genetic background. An important purpose of such studies is to develop algorithms assessing risk factors predictive for human and animal protothecosis.

Prototheca-ID is still a project in *statu nascendi* and will be further developed and refined by the authors in the next few years in order to enhance its data collection capacities and to improve and streamline its analytical performance and general functionality. Our near-term priority would be to install within the Prototheca-ID application, a module for accommodating and filtering the whole-genome sequence datasets, representing all *Prototheca* species. The *Prototheca* whole-genome sequencing project, initiated by our group in 2014, has already released a complete sequence of the *Prototheca wickerhamii* genome (19).

## Description of the authors

- Mikołaj Dziurzyński is a PhD student at the Department of Environmental Microbiology and Biotechnology at the University of Warsaw. He specializes in bioinformatics analysis of microbial genomes and metagenomes.
- Przemysław Decewicz is a postdoctoral researcher at the Institute of Microbiology at the University of Warsaw. His research interests include bioinformatics in phage and bacteria genomics.
- Mateusz Iskra is a PhD student, under the supervision of Dr Tomasz Jagielski, at the Department of Medical Microbiology at the University of Warsaw.
- Zofia Bakuła is a postdoctoral researcher at the Department of Medical Microbiology, University of Warsaw. In her research, she focuses on pathogens DNA in clinical management and epidemiology of infectious diseases.
- Tomasz Jagielski is the head of Department of Medical Microbiology at the University of Warsaw. He is one of the world’s top leaders in the field of *Prototheca* research.

## References

[1] Jagielski T. and Lagneau P.-E. (2007) Protothecosis. A pseudofungal infection. *J. Mycol. Med.*, 17, 261–270.

[2] Masuda M. , Jagielski T., Danesi P. et al. (2021) Protothecosis in dogs and cats - new research directions. *Mycopathologia*, 186, 143–152. 33206310 10.1007/s11046-020-00508-y

[3] Jagielski T. , Krukowski H., Bochniarz M. et al. (2019) Prevalence of *Prototheca* spp. on dairy farms in Poland - a cross-country study. *Microb. Biotechnol.*, 12, 556–566. 30891936 10.1111/1751-7915.13394 PMC6465227

[4] Ricchi M. , De Cicco C., Buzzini P. et al. (2013) First outbreak of bovine mastitis caused by *Prototheca blaschkeae*. *Vet. Microbiol.*, 162, 997–999. 23201242 10.1016/j.vetmic.2012.11.003

[5] Marques S. , Silva E., Kraft C. et al. (2008) Bovine mastitis associated with P*rototheca blaschkeae*. *J. Clin. Microbiol.*, 46, 1941–1945. 18434557 10.1128/JCM.00323-08 PMC2446845

[6] Ricchi M. , Goretti M., Branda E. et al. (2010) Molecular characterization of *Prototheca* strains isolated from Italian dairy herds. *J. Dairy Sci.*, 93, 4625–4631. 20854996 10.3168/jds.2010-3178

[7] Todd J.R. , Matsumoto T., Ueno R. et al. (2018) Medical phycology 2017. *Med. Mycol.*, 56, S188–S204. 29767780 10.1093/mmy/myx162

[8] Lass-Flörl C. and Mayr A. (2007) Human protothecosis. *Clin. Microbiol. Rev.*, 20, 230–242. 17428884 10.1128/CMR.00032-06 PMC1865593

[9] Jagielski T. , Gawor J., Bakuła Z. et al. (2018) CYTB as a new genetic marker for differentiation of *Prototheca* species. *J. Clin. Microbiol.*, 56, e00584–18. 10.1128/JCM.00584-18 PMC6156311 30068534

[10] Hirose N. , Nishimura K., Inoue-Sakamoto M. et al. (2013) Ribosomal internal transcribed spacer of *Prototheca wickerhamii* has characteristic structure useful for identification and genotyping. *PLoS One*, 8, e81223. 10.1371/journal.pone.0081223 PMC3842318 24312279

[11] Hirose N. , Hua Z., Kato Y. et al. (2018) Molecular characterization of *Prototheca* strains isolated in China revealed the first cases of protothecosis associated with *Prototheca zopfii* genotype 1. *Med. Mycol.*, 56, 279–287. 28525645 10.1093/mmy/myx039

[12] Jagielski T. , Bakuła Z., Gawor J. et al. (2019) The genus *Prototheca* (Trebouxiophyceae, Chlorophyta) revisited: implications from molecular taxonomic studies. *Algal Res.*, 43, 101639.

[13] Camacho C. , Coulouris G., Avagyan V. et al. (2009) BLAST+: architecture and applications. *BMC Bioinform.*, 10, 421. 10.1186/1471-2105-10-421 PMC2803857 20003500

[14] Katoh K. , Misawa K., Kuma K. et al. (2002) MAFFT: a novel method for rapid multiple sequence alignment based on fast Fourier transform. *Nucleic Acids Res.*, 30, 3059–3066. 12136088 10.1093/nar/gkf436 PMC135756

[15] Katoh K. and Standley D.M. (2013) MAFFT multiple sequence alignment software version 7: improvements in performance and usability. *Mol. Biol. Evol.*, 30, 772–780. 23329690 10.1093/molbev/mst010 PMC3603318

[16] Capella-Gutiérrez S. , Silla-Martínez J.M. and Gabaldón T. (2009) trimAl: a tool for automated alignment trimming in large-scale phylogenetic analyses. *Bioinformatics*, 25, 1972–1973. 19505945 10.1093/bioinformatics/btp348 PMC2712344

[17] Nguyen L.-T. , Schmidt H.A., von Haeseler A. et al. (2015) IQ-TREE: A fast and effective stochastic algorithm for estimating maximum-likelihood phylogenies. *Mol. Biol. Evol.*, 32, 268–274. 25371430 10.1093/molbev/msu300 PMC4271533

[18] Kalyaanamoorthy S. , Minh B.Q., Wong T.K.F. et al. (2017) ModelFinder: fast model selection for accurate phylogenetic estimates. *Nat. Methods*, 14, 587–589. 28481363 10.1038/nmeth.4285 PMC5453245

[19] Bakuła Z. , Siedlecki P., Gromadka R. et al. (2021) A first insight into the genome of *Prototheca wickerhamii*, a major causative agent of human protothecosis. *BMC Genomics*, 22, 168. 10.1186/s12864-021-07491-8 PMC7941945 33750287

